# Molecular Characterization and Function Analysis of the Vitellogenin Receptor from the Cotton Bollworm, *Helicoverpa armigera* (Hübner) (Lepidoptera, Noctuidae)

**DOI:** 10.1371/journal.pone.0155785

**Published:** 2016-05-18

**Authors:** Wanna Zhang, Long Ma, Haijun Xiao, Bingtang Xie, Guy Smagghe, Yuyuan Guo, Gemei Liang

**Affiliations:** 1 State Key Laboratory for Biology of Plant Diseases and Insect Pests, Institute of Plant Protection, Chinese Academy of Agricultural Sciences, Beijing, 100193, China; 2 Tea Research Institute, Chinese Academy of Agricultural Sciences, Hangzhou, 310008, China; 3 Institute of Entomology, Jiangxi Agricultural University, Nanchang, 330045, China; 4 Department of Crop Protection, Ghent University, Ghent, 9000, Belgium; Kansas State University, UNITED STATES

## Abstract

Developing oocytes accumulate plentiful yolk protein during oogenesis through receptor-mediated endocytosis. The vitellogenin receptor (VgR), belonging to the low-density lipoprotein receptor (LDLR) family, regulates the absorption of yolk protein. In this work, the full-length vitellogenin receptor (*HaVgR*) in the cotton bollworm *Helicoverpa armigera* was identified, encoding a 1817 residue protein. Sequence alignment revealed that the sequence of *Ha*VgR contained all of the conservative structural motifs of LDLR family members, and phylogenetic analysis indicated that *Ha*VgR had a high identity among Lepidoptera and was distinct from that of other insects. Consistent with other insects, *HaVgR* was specifically expressed in ovarian tissue. The developmental expression pattern showed that *HaVgR* was first transcribed in the newly metamorphosed female adults, reached a peak in 2-day-old adults and then declined. Western blot analysis also revealed an ovarian-specific and developing expression pattern, which was consistent with the *HaVgR* mRNA transcription. Moreover, RNAi-mediated *HaVgR* knockdown strongly reduced the VgR expression in both the mRNA and protein levels, which inhibited the yolk protein deposition in the ovaries, led to the dramatic accumulation of vitellogenin and the up-regulation of *HaVg* expression in hemolymph, and eventually resulted in a declined fecundity. Together, all of these findings demonstrate that *HaVgR* is a specific receptor in uptake and transportation of yolk protein for the maturation of oocytes and that it plays a critical role in female reproduction.

## Introduction

In insects, developing oocytes accumulate large amounts of vitellogenin (Vg) to meet the nutrient requirement for egg development. As the major yolk protein, Vg is primarily synthesized in the fat body for release into the circulatory system and is subsequently taken up by the competent oocytes [1]. This uptake is achieved by the vitellogenin receptor (VgR) which is located within clathrin-coated pits on the surface of growing competent oocytes [1–3]. Structural analysis of the deduced amino acid sequences from the insect VgRs revealed that VgRs are large membrane-bound proteins and belong to the low-density lipoprotein receptor (LDLR) superfamily [3, 4]. VgRs are composed of several common structural domains, including ligand-binding LDLR class A cysteine-rich repeats, epidermal growth factor (EGF)-like LDLR class B cysteine-rich repeats, repeats characterized by a YWXD motif that are assumed to form a β-propeller domain, an O-linked carbohydrate domain, a transmembrane domain, and a cytoplasmic tail containing an internalization signal [2, 5]. To date, the molecular characteristics of VgRs have been documented in many vertebrates, including chickens [6] and fish [7], and also studied in several insect species, for instance, *Aedes aegypti* [8, 9], *Solenopsis invicta* [10], *Bactrocera dorsalis* [11], cockroach [12, 13], *Nilaparvata lugens* [14] and some beneficial insects such as the silkworm and honey bee [15, 16]. Additionally, a yolk protein receptor functioning as the VgR has been well documented in the fruit fly [17]. These studies demonstrated that VgR mediated the Vg uptake during insect reproduction, thus, VgR could serve as a potential target for pest control [1, 2]. However, previous studies of VgR were mainly focused on the limited species of hygiene pests and beneficial insects, and few studies were focused on agricultural pests, particularly the notorious Lepidoptera moths.

The cotton bollworm, *Helicoverpa armigera*, is one of the most serious pests of cotton, corn, vegetables and many other crops. In recent years transgenic cotton expressing the Cry 1Ac gene from *Bacillus thuringiensis* (Bt) has controlled this pest effectively [18, 19]. However, *H*. *armigera* has evolved resistance to Cry1Ac and several resistant individuals have been detected in both the laboratory and field [20, 21]. This has resulted in a sense of urgency in the development of novel pest management strategies. Obviously, reproduction is the basis for the exponential growth of a pest population. The synthesis, secretion and uptake of Vg are important for the reproductive development in insects. A better understanding of candidate genes regulating insect reproduction can provide potential approaches for pest control. Previously, we have characterized the *H*. *armigera Vg* at both the biochemical and molecular levels, including gene cloning and sequence analysis [22]. Despite the lack of a sequenced genome, the physiology, metabolism and reproduction of *H*. *armigera* have been studied intensively because of its devastating nature. Considering the function of yolk protein in the reproduction of insects, the current study determined the full length *HaVgR* cDNA. The basic molecular and structural characteristics of *Ha*VgR were analyzed and compared with those from other insects. In addition, we reported the tissue- and developmental profile of *HaVgR* by quantitative PCR (qPCR) and a western blot assay. Finally, we used RNA interference to verify the function of *HaVgR* in the ovarian development.

## Materials and Methods

### Ethnics statement

Our study used New Zealand female rabbits to generate polyclonal antibodies. All experimental procedures were conducted in conformity with institutional guidelines for the care and use of laboratory animals. Our experimental design and procedures were approved by the Animal Care and Use Committee of the Chinese Academy of Agricultural Sciences, Beijing, China. During the experiment, the rabbits were maintained individually in large cages with sufficient feed and water, and all protocols were performed in conformity with ethical guidelines to minimize pain and discomfort to the animals.

### Insects raring and samples collection

*H*. *armigera* was reared in the laboratory on an artificial diet at 27 ± 2°C, 75± 10% RH and a photoperiod of 14: 10 (L: D). The larvae were reared on an artificial diet in the 24-well plate, and they were transferred into 25-ml glass tubes containing an artificial diet at the fifth instars for pupating (one larvae per tube). After emergence, the adults were placed in cages (30 cm × 60 cm × 30 cm) for oviposition and supplied with 10% sugar solution.

For developmental expression analysis, the freshly emerged female adults were collected daily until death; old female pupae and male adults were also collected. The samples were frozen immediately in liquid nitrogen and subsequently stored at -80°C for further experiment. For tissue-specific expression profiles, tissues (including ovary, epidermis, midgut, fat body and malpighian tubules) and segment (head) were dissected from 4-day-old female adults in phosphate-buffered saline (PBS), these samples were frozen immediately in liquid nitrogen and stored at -80°C until RNA isolation.

### The extraction of RNA and cDNA synthesis

Total RNAs were extracted from the *H*. *armigera* tissues or the whole body using Trizol reagent (Invitrogen, Carlsbad, CA). The RNA sample was dissolved in 20 μl of diethylpyrocarbonate (DEPC)-treated H_2_O and evaluated at an absorbance ratio of OD 260/280 (1.8–2.1) using a NanoVue spectrophotometer (GE-Healthcare, Germany). The RNA integrity was confirmed using 1% agarose gel electrophoresis. After digestion of residual genomic DNA with DNase I (Promega), 2 μg of the total RNA samples were reverse transcribed in 20 μl reaction mixtures using the Fast Quant RT kit (TIANGEN, Beijing, China).

### Molecular cloning

Two VgR cDNA fragments were identified in the transcriptome of *H*. *armigera* (unpublished data). The sequences of these two regions were substantiated by PCR amplification using the primers listed in S1 Table. PCR reactions were performed as follows: one cycle pre-denaturing at 94°C for 4 min, 35 cycles of 95°C for 30 sec, 58°C for 30 sec, and 72°C for 2 min; and then 72°C for 10 min for elongation. The PCR products were gel purified, subcloned into the T3 vector (TransGen Biotech, Beijing, China) and transformed into *Escherichia coli* Trans1T1-competent cells (TransGen Biotech, Beijing, China). Positive clones were confirmed by PCR and sequenced. To obtain the full length cDNA sequence of *HaVgR* gene, a SMART^TM^ RACE (rapid amplification of cDNA ends) cDNA amplification kit (Clontech, Mountain View, CA) was used to amplify the 5'end and 3'end. Touchdown PCR was performed using gene-specific primers (*HaVgR* 5RACE and *HaVgR* 3RACE) and the universal primer mix (UPM) with the following protocol: five cycles of 30 sec at 94°C and 3 min at 72°C; five cycles of 30 sec at 94°C, 30 sec at 70°C and 3 min at 72°C, followed by 30 cycles of 30 s at 94°C, 30 sec at 68°C and 3 min at 72°C; and a final extension at 72°C for 10 min. The RACE products were purified and sequenced as described above.

### Sequence comparisons and phylogenetic analysis

Sequences were analyzed according to nucleotide and protein database using the BLAST website (http://www.ncbi.nlm.nih.gov/BLAST/). The coding sequence was predicted by the NCBI open reading frame (ORF) finder (http://www.ncbi.nlm.nih.gov/gorf/orfig.cgi.). The putative protein sequence was compared against the non-redundant GenBank protein database using BLASTP. Tools available from the ExPASy proteomics server (http://www.expasy.org) were used to determine putative molecular weights and isoelectric points (Compute pI/Mw tool). The signal peptide position and the transmembrane helices were predicted with Signal IP 4.1 Server (http://www.cbs.dtu.dk/services/SignalIP/) and TMHMM 2.0 Server (http://www.cbs.dtu.dk/services/TMHMM-2.0/), respectively. The SMART program (http://smart.embl-heidelberg.de/) was used to identify the conserved domains. The percent identity of the amino acid sequences was calculated using Vector NTI. Finally, a phylogenetic tree was constructed with MEGA 5.0 using the neighbor-joining method with a p-distance model and a pairwise deletion of gaps. Bootstrap support was assessed by a boot strap procedure based on 1000 replicates [23].

### Temporal expression patterns and tissue specific expression of *HaVgR*

The qPCR analysis was carried out on an ABI 7500 Real-Time system with *Premix Ex Taq*^TM^ Kit (TaKaRa, Tokyo, Japan) according to the manufacturer's instructions. Specific primers used for the qPCR were designed using Primer 3 (v.0.4.0) (http://bioinfo.ut.ee/primer3-0.4.0/) and listed in S1 Table. Two reference genes, *β-actin* (Accession no. EU527017) and *Gapdh* (Accession no. JF417983), were used as internal controls. To make sure that the amplification efficiencies of target genes and reference genes are approximately equal, the efficiency of each primer pair was analyzed by constructing a standard curve with ten-fold cDNA dilution series (S1 Fig). The qPCR amplifications were performed in a total volume of 20 μl, containing 1 μl of cDNA, 10 μl of *Premix Ex Taq*^TM^ (2×), 0.4 μl of each primer (10 μM), 0.8 μl of Probe, 0.4 μl of ROX Reference Dye II (50×) and 7.0 μl of ddH_2_O. The qPCR program consisted of one cycle of 95°C for 30 sec, followed by 40 cycles of 95°C for 5 sec, 60°C for 30 sec. To ensure reliability, each reaction for each sample was performed in four biological replications, each with three technical replicates; each reaction included negative controls without template. The comparative Ct method (2^-ΔΔCt^) was used to calculate the relative transcript level [24].

### Expression of recombinants and polyclonal antibody production for *Ha*VgR and *Ha*Vg

Two cDNA fragments, partial sequence of *HaVgR* (from 169 to 1026 bp, 57–342 aa) and *HaVg* (from 2103 to 3000 bp, 701–1000 aa), were amplified with primer pairs (VgRP-F and VgRP-R, VgP-F and VgP-R, S1 Table) containing the restriction sites for *BamH I* and *Xho I*, respectively. The PCR product was subcloned into the pGEM-T-Easy vector (Promega). The target fragment was digested with *BamH I* and *Xho I* and then cloned into pET-30a (+) vector (Novagen, Germany). The recombinant *Ha*VgR and *Ha*Vg protein were expressed in BL21 (DE3) competent cells induced by 0.6 mM IPTG. After purification by the NTA-Ni^2+^-His Bind Resin (GE-Healthcare, Germany) (S2 Fig), the *Ha*VgR and *Ha*Vg proteins were used to immunize rabbits, which in turn produced polyclonal antibodies as described previously [25]. The sera of the immunized rabbits were collected as anti-*Ha*VgR sera and anti-*Ha*Vg sera. The antiserum was purified and the specificity of the antiserum was examined by immunoblotting.

### Protein extraction and western blot

Tissues or whole bodies of *H*. *armigera* were homogenized in lysis buffer (8 M urea, 4% chaps, 40 mM Tris–HCl, 5 mM EDTA, 1 mM PMSF and 10 mM DTT, pH 8.0) containing a mixture of protease inhibitors (Roche, Switzerland). The total protein concentrations were determined by a Bio-Rad protein assay using bovine serum albumin (BSA) as the standard (Bradford, 1976). The samples were then diluted with loading buffer to obtain equal amounts of the total proteins. After the proteins were separated by 12% (w/v) SDS–PAGE, they were transferred onto nitrocellulose (NC) membrane blotting filters at a contrast 100 V for 1 h at 4°C. Membranes were blocked with 5% (w/v) skimmed milk in PBST (35 mM NaCl, 2 mM KCl, 10 mM Na_2_HPO_4_, 1.7 mM KH_2_PO_4_, PH 7.4, 0.05% Tween-20) at 4°C overnight, and then the membranes were washed with three times in PBST for 15 min per wash. After blocking, the membrane was incubated with the *H*. *armigera β*-actin antibody (1: 2000) and either the *Ha*Vg antibody (1: 4000) or the *Ha*VgR antibody (1: 5000) for 1 h at room temperature. After three washes with PBST (15 min each), the membrane was incubated for 1 h at room temperature with goat anti-rabbit IgG HRP-linked secondary antibody (Sigma, St. Louis, MO, USA) diluted to 1:10,000 with PBST. The immunoreactivity was visualized using an enhanced electrochemiluminescence (ECL) detection kit (TransGen, Beijing, China) and photographed by Image Quant LAS4000 mini (GE-Healthcare, Germany).

### Synthesis of dsRNA and RNA interference

To synthesize the double-stranded RNA (dsRNA), two primers with a T7 promoter (T7 VgR-F, T7 VgR-R, S1 Table) were designed to amplify a 534 bp fragment of *Ha*VgR (from 3211 to 3744). The amplification protocol consisted of 35 cycles of 95°C for 30 sec, 55°C for 30 sec and 72°C for 40 sec, with a final extension of 72°C for 10 min. The PCR products were then excised from the ethidium bromide-stained gel and purified using a DNA purification kit (Tiangen, Beijing, China). The dsRNA was synthesized in vitro using a HiScribeTM T7 Transcription Kit system (New England BioLabs, Ipswich, MA) and finally re-suspended in DEPC water. Additionally, a 500 bp segment GFP (ACY56286) dsRNA was synthesized as negative control. The purity of the dsRNA was checked on a 1.0% agarose gel.

The newly emerged female adults were injected in the abdomen with 1 μl dsRNA of *Ha*VgR (5 μg) using a 5 μl-microsyringe (Hamilton, Bonaduz, Switzerland). The injection point was sealed immediately with geoline. In addition, two parallel controls were performed, each containing an equivalent volume of dsGFP (5 μg) and DEPC water. Each treatment included 90 individuals with three replicates. To calculate the RNAi efficiency by qPCR and western blot, 10 moths were randomly selected at 24 h, 48 h and 72 h after the injection. To examine the effect of RNAi on fecundity, 30 treated females were chosen for an oviposition bioassay, each of which was paired with two untreated males in one plastic cup (8 cm in diameter, 10 cm high). The plastic cups covered with one layer of 10 cm×10 cm gauze were held in the same condition as above. Cotton wicks were placed on the gauze to supply 10% sugar solution, and both the gauze and the cotton wick were changed daily to count the number of eggs laid. Additionally, in each treatment, the ovarian phenotypes of 4-day-old female moths were observed after dissection under a stereomicroscope (Olympus BX61, Tokyo, Japan). Briefly, the numbers of follicles at each developmental stage were recorded to calculate the proportion of mature follicles, and the length of the mature oocyte was measured. Observations of each treatment included 15 females, and the lengths of mature follicles in each female were measured.

### Data analysis

All data obtained from the studies were presented as means ± SE. The results were analyzed by one-way analysis of variance (ANOVA), followed by a least significant difference test (LSD) for mean comparison. The proportion of mature follicles were arcsine transformed before ANOVA to meet the assumptions of normality. Statistical difference of expression levels between females and males were tested by Student's t-test. All statistical analysis was performed with SAS 9.20 software (SAS Institute, Cary, NC) at *P* < 0.05 level of significance.

## Results

### Sequence and structural analysis of the *HaVgR*

The full-length *HaVgR* cDNA was 5949 bp, comprising a 188 bp 5' untranslated region (UTR), a 5454 bp open reading frame (ORF) encoding 1817 residues and a 307 bp 3' UTR (Accession no. AGF33811.1). The analysis of the deduced amino acid sequence revealed that a signal peptide with 16 amino acids (MKYQSIILILCVAACS) was located at the N-terminal of *Ha*VgR. The deduced protein sequence of *Ha*VgR predicted a protein with a molecular mass of 203.2 kDa and a pI value of 5.08. Analyses of the *Ha*VgR protein sequence indicated that, similar to other insect VgRs, it contained all of the features that are typical of the LDLR family. *Ha*VgR exhibited two ligand-binding domains (LBDs) with four class A (LDLR_A_) cysteine-rich repeats in the first domain (LBD1) and seven repeats in the second domain (LBD2). Each repeat contained six cysteine residues and each LDLR_A_ was followed by an epidermal growth factor (EGF)-like domain. The first EGF-like domain contained a calcium-binding domain, two YWXD domains and a LDLR repeat class B (LDLR_B_), whereas only a single EGF-like domain was found in the second one. Following the second EGF-like domain, a transmembrane domain spanning amino acids 1691–1715 and a cytoplasmic domain spanning amino acids 1716–1817 were predicted using the TMHMM sever v. 2.0 and ExPASY (S3 Fig).

**Fig 1:**
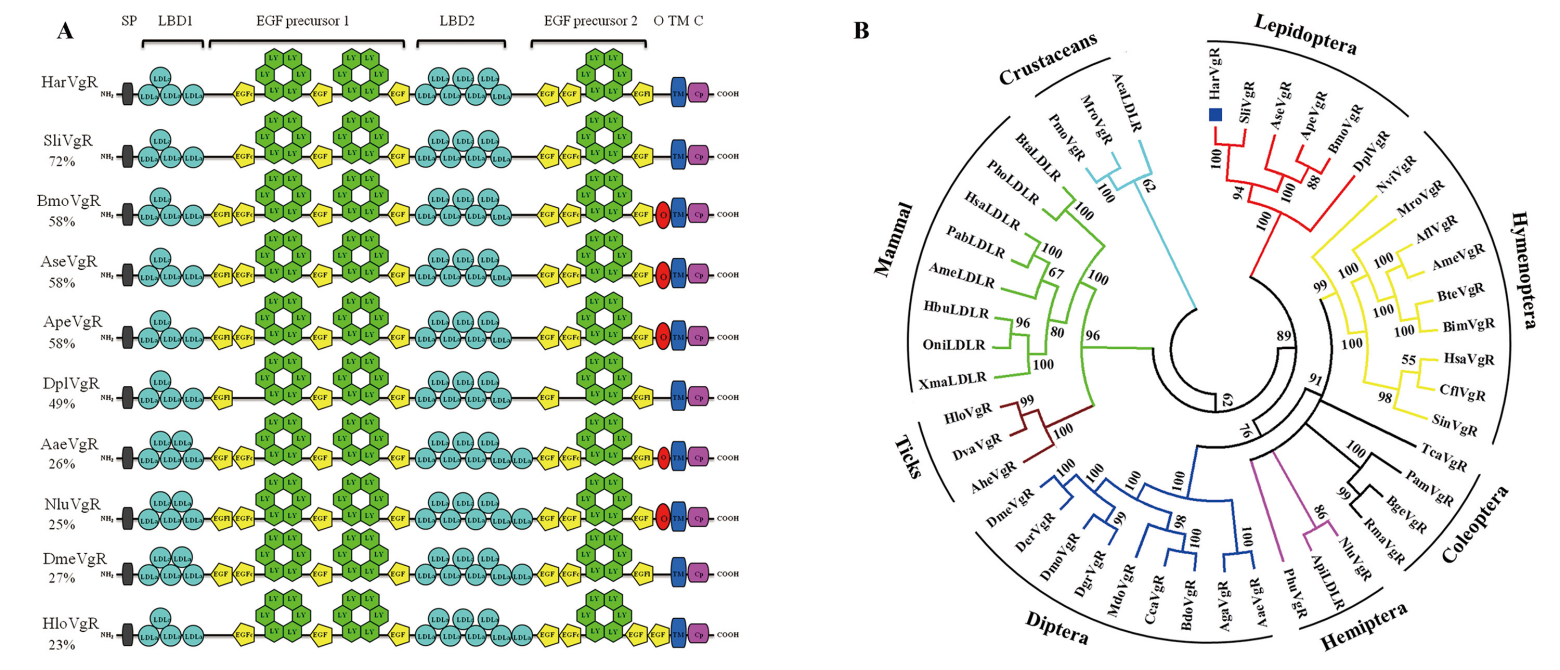
**Diagrammatic comparison of primary structure of *Ha*VgR with other insects (A) and the molecular phylogenetic tree constructed based on the sequence of vitellogenin receptor (VgR) and low density lipoprotein receptors (B).** EGFc indicate non-calcium binding. The YWXD containing repeats that form b-propeller domains are labeled LY. The percentages on the left indicate overall identity of each protein compared to *Ha*VgR. SP, signal peptide; LBD, lipid binding domain; O, potential O-linked sugar domain; TM, transmembrane domain; C, cytoplasmic domain. The protein names and accession numbers used in this analysis are listed in S2 Table.

When compared to other insect VgRs, the *Ha*VgR protein showed similarities of 72%, 58%, 58%, 58% and 49% with *S*. *litura*, *A*. *selene*, *B*. *mori*, *A*. *pernyi* and *D*. *plexippus* respectively, in a sequence alignment. The *Ha*VgR protein also revealed identities of 28%, 27%, 26%, 25% and 23% with *P*. *americana*, *D*. *melanogaster*, *A*. *aegypti*, *N*. *lugens* and *H*. *longicornis*, respectively. The Lepidopteran insects had 11 LDLR_A_ repeats, while 12 or 13 repeats occurred in the VgRs of other insects (Fig 1A).

A phylogenetic tree was constructed based on the distances of amino acid sequences comparing *H*. *armigera* and other insect VgRs or LDLRs (Fig 1B). The dendrogram clustered VgRs from Lepidopteran insects in one branch, suggesting that they all had relatively high amino acid sequence identity. Typically, the Hymenopteran VgRs closely clustered with the Coleopteran VgRs, suggesting that these VgRs shared a closer ancestry than other insect VgRs. The present phylogenetic analysis also revealed that the tick VgRs were more closely related to the mammal LDLRs than the insect VgRs, which is interesting because in general, VgRs from insect species had a closer ancestry than VgRs from crustacean and mammal species.

### Tissue specific and developmental expression pattern of *HaVgR*

**Fig 2:**
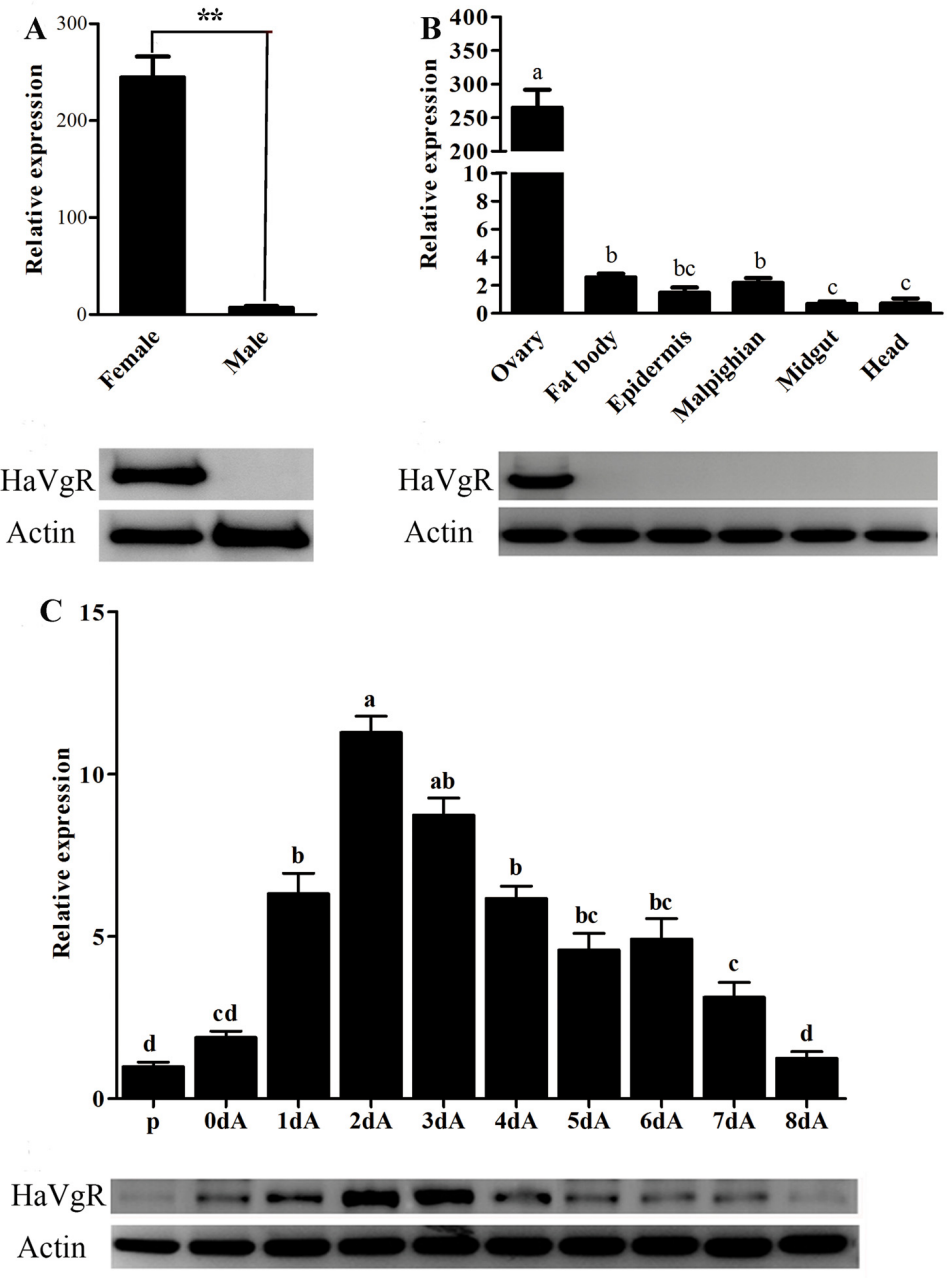
qRT-PCR and western blot analysis of sex-, tissue-specific and development expression patterns of *HaVgR*. A: Sex-specific expression of *Ha*VgR. The total RNA samples were extracted from abdomens of female and male adults. B: Tissue-specific expression of *Ha*VgR. The total RNA samples were extracted from various of female tissues and segment: ovary, epidermis, midgut, fat body, malpighian tubules and head. C: Development expression patterns of *Ha*VgR. Samples were extracted from abdomens of the 10-days-old pupae (P), 0-day-old adults (0dA), 1-day-old adults (1dA), 2-days-old adults (2dA), 3-days-old adults (3dA), 4-days-old adults (4dA), 5-days-old adults (5dA), 6-days-old adults (6dA)), 7-days-old (7dA), 8-days-old (8dA). The bars represent the average (±SE) of biological repeats. Different letters indicate significant difference (*P* <0.05). ** indicate significant (*P* <0.01) differences between two groups.

**Fig 3:**
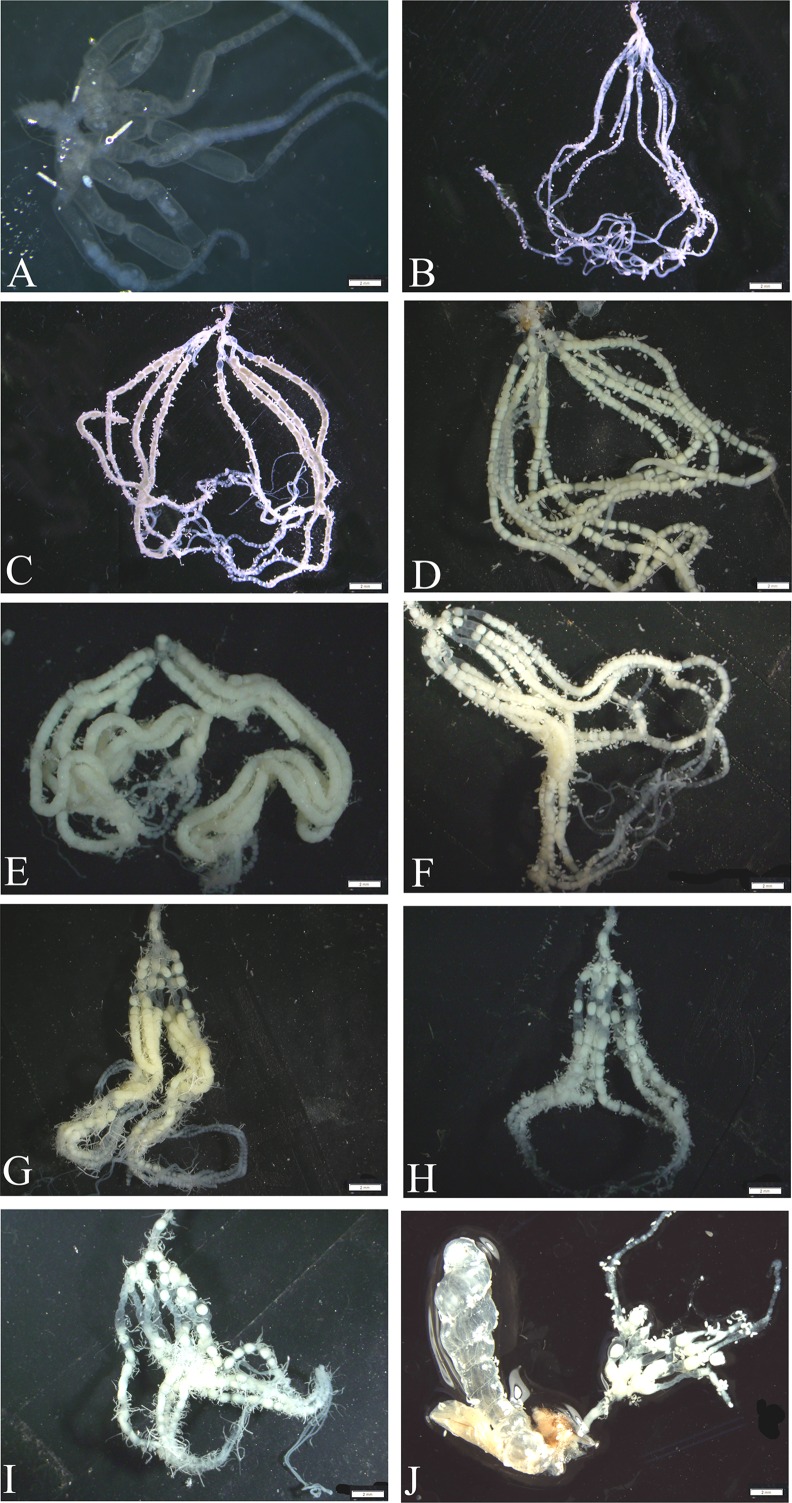
The ovarian development of *H*. *armigera*. The ovarian images of pupae, 0-day-old female to 8-day-old female (A-J) were observed. Bar: A-J = 2 mm.

The qPCR analysis with the cDNA template from the abdomen of male and female adults demonstrated that *HaVgR* mRNA was expressed in female abdomen (Fig 2A). Additionally, *HaVgR* was specifically expressed in the ovarian tissues (Fig 2B). Quantification by qPCR confirmed that the expression level of *HaVgR* in the ovary was significantly higher than that of other tissues (*F* = 12.29, *df* = 6, 21, *P* < 0.0001). Furthermore, the developmental profile of *HaVgR* transcription showed that the VgR expression in the ovary was detected in the newly emerged females and it reached a maximum level in the 2-day-old individuals (Fig 2C). Subsequently, the expression level decreased significantly in 3-day-old female adults compared with that in the 2-day-old moths (*F* = 12.305, *df* = 1,14, *P* = 0.003), and it maintained the low level during the late adult period (Fig 2C). To measure the sex- and tissue-specific expression profiles and developmental expression patterns of *Ha*VgR, western blot analysis was performed with the total protein extracted from different tissues and insect whole bodies. It was shown that *Ha*VgR protein dection was unique to the female ovary, while no protein band was detected in other tissues of female or in the male, consistent with the qPCR results (Fig 2B). Additionally, the developmental pattern of *Ha*VgR protein showed that it first appeared on the newly emerged day, and the expression level increased with the insect development with the maximum level in the 2-day-old and 3-day-old adults (Fig 2C). The expression pattern was consistent with the ovarian development (Fig 3).

### Effects of *Ha*VgR-dsRNA treatment in *H*. *armigera*

**Fig 4:**
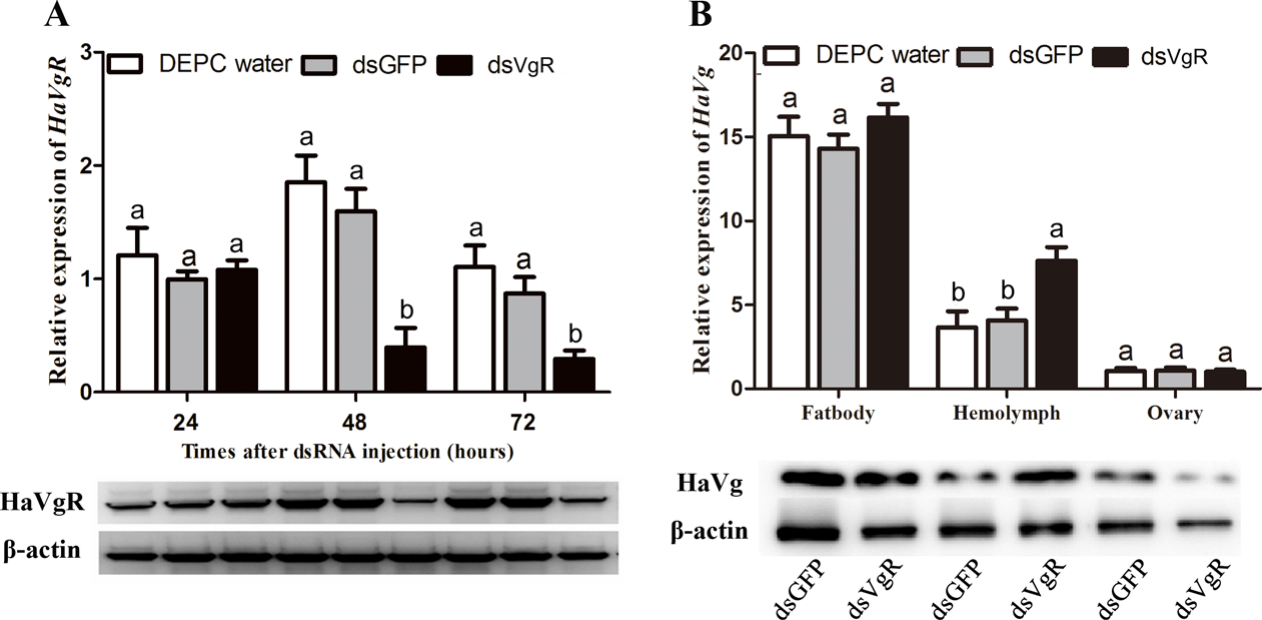
Detection of mRNA and protein level in RNA-interference-treated insects. (A): The expressions levels of *Ha*VgR were determine by qPCR and western blot after injection of dsVgR 24, 48 and 72 h. (B): The expression levels of *Ha*Vg were analyzed by qPCR and western blot after injection of dsVgR 48 h. The dsGFP treatment group was used as a negative control and DEPC water was used as a blank control. The bars represent the average (±SE) of biological repeats. Different letters indicate significant difference (*P* <0.05).

To investigate the efficiency of the RNAi, *HaVgR* mRNA levels were measured by qPCR. The *HaVgR* transcript levels in dsVgR-injected female were significantly decreased by 78.6% (48 h after treatment) and 73.5% (72 h) compared to the dsGFP treated group (48 h: *F* = 14.434, *df* = 1,10, *P* = 0.002; 72 h: *F* = 8.436, *df* = 1, 10, *P* = 0.009). These results were confirmed by the western blot analysis that the HaVgR protein level in dsVgR-injected females was also significantly decreased (Fig 4A). Additionally, there was no obvious influence on the transcription level of *HaVg* mRNA in either the ovary or fat body after dsVgR injection, compared with the dsGFP at the time of 48 h after treatment (ovary: *F* = 0.109, *df* = 1, 10, *P* = 0.898; fat body: *F* = 2.536, *df* = 1,10, *P* = 0.143). The expression level of *HaVg* mRNA in hemolymph was up-regulated when the individuals were treated with dsVgR (*F* = 11.001, *df* = 1,10, *P* = 0.008) (Fig 4B). Although there were no significant changes in the *HaVg* mRNA level after injection with dsVgR, the content of *Ha*Vg protein in the ovary dropped significantly along with the evident accumulation of the *Ha*Vg protein in the hemolymph (Fig 4B).

**Fig 5:**
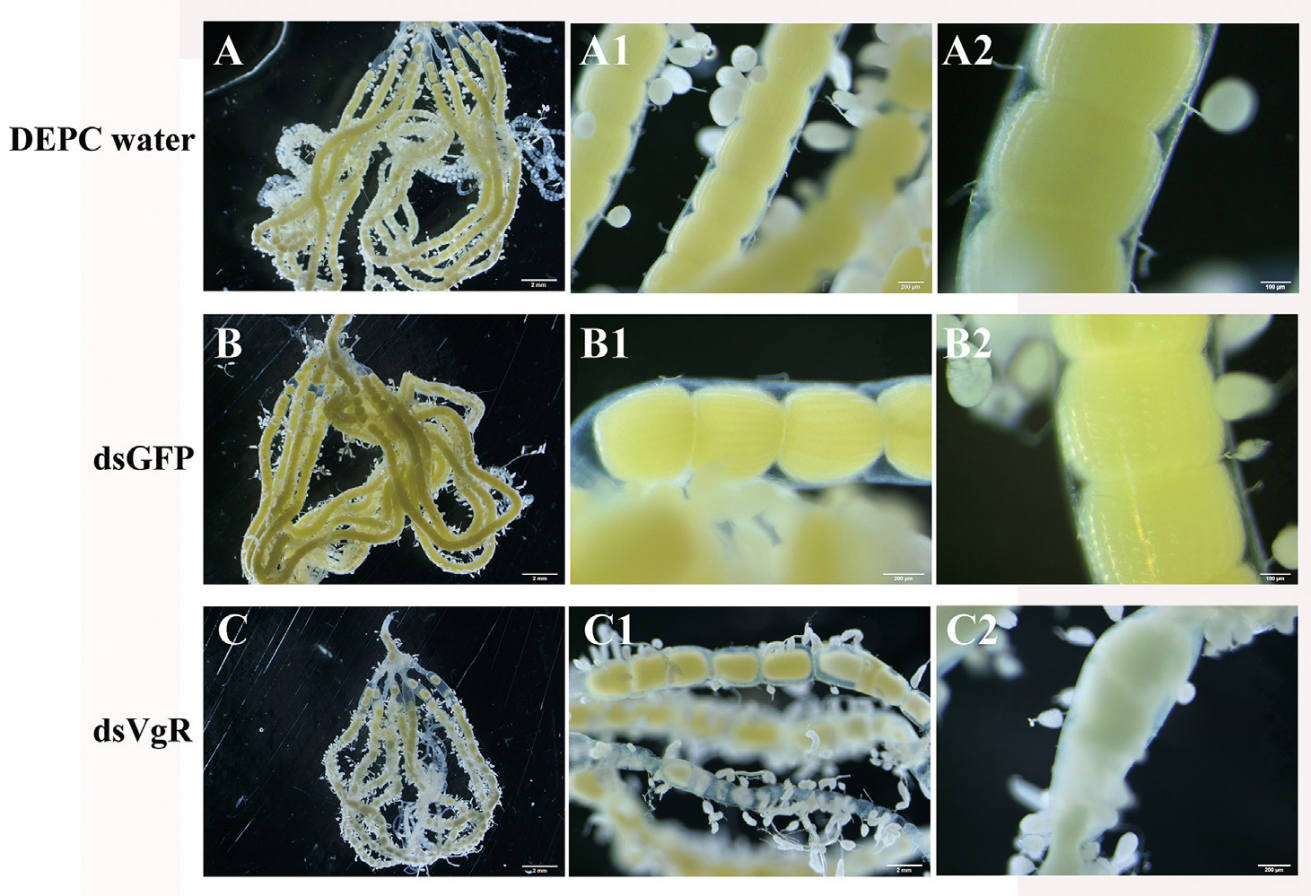
Ovary development was evaluated after RNAi-mediated knockdown of HaVgR. Ovaries were dissected and photographed under dissection microscopy at days 4 post-injection, the dsGFP was injected as a negative control and DEPC water was injected as the blank control. DEPC water (A-A2), dsGFP (B-B2) and dsVgR (C-C2).

To evaluate the effect of *Ha*VgR silence on the oviposition and ovary development, the number of eggs was documented daily, and the ovaries were also dissected from both the treated and control female adults at four days post-treatment. Moths injected with 5 μg of dsVgR exhibited an 81%-decrease in oviposition compared with those treated with DEPC water (Table 1). The dissection of ovary showed a decrease in yolk protein deposition in the *Ha*VgR silencing moths, exhibiting a small degree of yolk uptake in oocytes (Fig 5). Moreover, the number of follicles, especially the mature follicles, in individuals treated with dsVgR was less than that in the controls (*F* = 40.85, *df* = 1, 28, *P* < 0.001). The proportion of mature follicles of moths treated with dsVgR was significantly lower than that of moths treated with dsGFP (*F* = 9.115, *df* = 1, 28, *P* = 0.007), and the same trend was observed in the length of mature follicles (*F* = 21.00, *df* = 1, 28, *P* < 0.001). The proportion of mature follicles (*F* = 0.456, *df* = 1, 28, *P* = 0.51) and the length of mature follicle (*F* = 1.279, *df* = 1, 28, *P* = 0.27) were observed no significant difference between the two controls (dsGFP and DEPC water treated), respectively (Table 1).

**Table 1:** Effect of HaVgR-dsRNA on oviposition of *H*. *armigera*.

Treatment	dsGFP	DEPC water	dsVgR
**Total number of eggs laid**	576.25±3.12 a	595.36±4.15 a	110.45±5.00 b
**Number of follicles**	890.37±30.25 a	998.45±88.76 a	456.67±45.33b
**Proportion of mature follicles (%)**	57.32±4.71 a	54.79±1.92 a	36.13±3.72 b
**Length of mature follicles (μm)**	405±2.10 a	391±1.50 a	295±1.90 b

Note: Data are expressed as mean numbers of eggs deposited per female ± SE. Different letters after the numbers indicate significant differences at *P* < 0.05. The number of follicles and the proportion and length of mature follicles were recorded in 4-day-old female moths of each treatment.

### Discussion

The recent cloning and sequence analysis of several insect VgRs have brought the study of insect reproduction to a new plane. In our study, the putative *HaVgR* was cloned and characterized as the LDLR homologues from the cotton bollworm, *H*. *armigera*. This is the first report that used RNA interference to demonstrate the role of *HaVgR* on the oviposition and ovary development of *H*. *armigera*. The results clearly showed that silencing the *HaVgR* gene inhibited the ovary development and effectively reduced the fecundity of *H*. *armigera* females by disturbing the uptake of Vg.

The *Ha*VgR displayed high sequence similarity to VgRs from insect, crustacean and vertebrate [1, 2]. Analysis of domain conservation between *Ha*VgR and other insect VgRs suggests *Ha*VgR to be a member of the LDLR family bearing five highly conserved arrangements of modular elements (Fig 1). In particular, there is a striking homology between *Ha*VgR and other Lepidopteran VgRs, such as *S*. *litura*, *B*. *mori*, *A*. *selene* and *A*. *pernyi*. This may be a result of the high similarity of their ligands. In schematic comparison, *Ha*VgR has four cysteine-rich LDLR_A_ repeats in the first ligand-binding site and seven in the second, which is unique in Lepidoptera [15, 26, 27, 28]. This structure differed from other insect orders, which generally included a total of 13 LDLR repeats in five- and eight- repeats arrangements (Fig 1A) [2]. The arrangement in Lepidoptera was also different from the classical LDLRs, which had a single seven-repeat domain [29], and different from vertebrate VgRs [30] and VLDLRs [31], both of which had a single eight-repeat domain. Additionally, the *Ha*VgR included two EGF-like domains (EGF precursor 1 and EGF precursor 2, Fig 1A). Similar to the VgR from *S*. *litura* [27], *S*. *invicta* [10] and *D*. *melanogaster* [32], no O-link sugar domain appeared in *Ha*VgR, which was different from the *Aa*VgR and *Nlu*VgR, indicating that this domain was not unique among invertebrate VgRs. Besides, a NPXY motif, which has been proved to be necessary and sufficient for the internalization of LDLR, was also found in the cytoplasmic tail of *Ha*VgR (S3 Fig).

As reported in other insects [3, 14, 27], *HaVgR* was also specifically expressed in the ovary (Fig 2B), indicating that VgR was one ovary-specific member of the LDLR superfamily and participated in reproduction. However, in *A*. *mellifera*, the extra-ovarian *VgR* expression was detected not only in the hypopharyngeal gland of honeybee workers but also in other tissues [16, 33]. The detection of VgR mRNA in tissues other than the ovary could be related to the pleiotropic roles of its ligand Vg in the social life of the bees, including the regulation of queen/worker longevity and the juvenile hormone titer [34, 35]. Similarly, in some species of vertebrates, the VgR transcript was also detected in other tissues, such as heart, liver, brain, muscle and even male testis [30, 36, 37]. Thus from the tissue-expression analysis, it is speculated that the diverse expression of VgR may due to the alternative functions of their ligands.

The role of VgR in the deposition of the yolk protein was undisputed, and the dependence of VgRs expression upon ovarian development patterns had previously been studied in series of insects [3]. In the current study, *HaVgR* was expressed along all stages of the ovarian development, reaching the peak in the early vitellogenic period (2 days after emergence) (Fig 2C), which was closely correlated with the ovarian development (Fig 3). This expression pattern was similar to the observation in other insects. In *S*. *invicta*, VgR transcript of reproductively active queens was in lower levels than that of virgin alate females [10], and the expression analysis in cockroaches revealed that the highest expression level of VgR orthologs were observed in the immature ovaries of nymphs as well as in the ovary containing early previtellogenic oocytes [12, 13, 38]. Additionally, this expression pattern also resembled that of the VgR transcript reported from the non-insect species. For instance, in chicken and rainbow trout, VgR transcript levels were high in the early vitellogenic periods, yet low detectable VgR signals were present among fully vitellogenic oocytes [6, 7]. However, in the mosquito *A*. *aegypti*, translation of the VgR began during the previtellogenic development, continued with dramatic rise during the vitellogenic period, and peaked at 24–30 h post blood meal; the expression level was correlated with the ecdysteroid titer in females following the blood meal [9]. In ticks, it was reported that the VgR message was only expressed in adult females that had fed to repletion, not in unfed or partially fed females [39]. The expression pattern also showed that the *Ha*VgR expression was up-regulated in the period of sexual maturation (0–2 days after emergence), but quickly declined in the phase of egg maturation, which was ahead of the decline of Vg mRNA [22]. Therefore, it is hypothesized that the over-expression of *Ha*VgR works as a precondition for the effective endocytosis of Vgs in female adults. Additionally, the decrease in VgR transcript levels in mature oocytes was interpreted as the generation of functional receptor before maturation of oocytes. Overall, in combination with the ovarian phenotypes (Fig 3), we concluded that the yolk deposition was mainly mediated by the VgR protein and the arrival of peak expression was determined by the yolk deposition.

Gene silencing through RNAi is a powerful tool to explore gene function [40], nevertheless, RNAi has many times proven to be difficult to achieve in Lepidoptera [41]. When considering that successful experiments have been reported in one life stage of insects and not in others [42, 43, 44], newly emerged females and 5 μg exogenous dsVgR per moth were selected as the appropriate stage and the optimal dose for microinjection. After dsVgR treatment, the mRNA expression of *HaVgR* decreased progressively, and the affection lasted for at least 72 h after injection (Fig 4A). As expected, the levels of *Ha*VgR protein in the ovary of the dsVgR-treated females were much lower than in the controls. The calculated sensitivity to RNAi (amount of dsRNA administered per mg tissue required to achieve silencing) in our RNAi is 18.5 ng/mg which appears to be a low dose. Such low concentration was chosen at which the efficiency was maintained but at a minimal risk of non-specific effects. In *Laphygma exigua*, the common levels of high silencing was achieved with concentrations of dsRNA at 0.3–0.5 μg/mg [45].

The primary function of Vg was to provide a pool of amino acids for the embryo, it also functioned as a carrier of carbohydrate, lipids, phosphates, vitamins, metals and hormones [46]. However, the uptake of Vg was along with the necessity for specifically binding a VgR which was involved in the delivery of Vg into the mature yolk bodies [2]. Similar to the previous studies in *S*. *litura* [27], *N*. *lugens* [14], *B*. *germanica* [12] and *S*. *invicta* [10], when *Ha*VgR was silenced, the developing oocytes failed to absorb Vg and a mass of Vg accumulated in the hemolymph, which consequently led to the little Vg deposited in the ovary (Fig 4B). This was consistent with the anatomic ovarian phenotype that less mature oocytes were deposited in ovaries after dsVgR treatment (Fig 5), indicating that the ovarian development was significantly inhibited. However, no corresponding decrease was observed in *Vg* mRNA transcription (Fig 4B). Additionally, the oviposition rate decreased significantly (81%) compared to the DEPC water injected group (Table 1). Similar result was reported in *B*. *germanica*, the treatment of VgR-dsRNA disturbed the yolk deposition and reduced the fecundity [12]. In the ticks *A*. *hebraeum*, *N*. *lugens* and *B*. *dorsalis*, female adults injected with a VgR-dsRNA probe experienced a significant delay in ovary development and were postponed to oviposit relative to the control [11, 14, 40]. Moreover, the VgR mutant of the silkworm failed to produce eggs because this mutation was lethal in embryos [47]. In terms of the VgR expression profiles and the influence on fecundity, it was reasonable to conclude that VgR participated in transporting yolk proteins for egg formation and was indispensable for insect reproduction.

In conclusion, our study demonstrates that the *HaVgR* gene serves as a potential target for effective pest control, and our findings would enrich the understanding of insects VgRs that are highly conserved in both structure and expression patterns. The determination of the full-length sequence of the *HaVgR* gene along with its molecular characteristics and the expression pattern represented the first step to understanding the molecular mechanisms of oogenesis in *H*. *armigera*. The function of *Ha*VgR was further analyzed by RNAi. It was apparent that *Ha*VgR was necessary to stimulate yolk uptake, critical for regulating the yolk protein deposition, and played an indispensable role in oviposition. However, it is still unclear how *Ha*VgR interacts with *Ha*Vg to regulate oogenesis under the influence of hormones. More work is required to explore the physiological mechanism of reproduction in *H*. *armigera* to provide an effective strategy for pest management.

## Supporting Information

S1 Fig Amplification efficiency curve of *H*. *armiger*a genes used in this experiment determined by qRT-PCR. The Ct was calculated for each cDNA dilution. The amplification efficiency of *β-actin*, *Gapdh*, *HaVgR* and *HaVg* was 107.27%, 101.43%, 96.49% and 103.04%, respectively.
(TIF)

S2 Fig **SDS-PAGE analyses showing the expression and purification of recombinant HaVgR (A) and HaVg (B)**. M: Protein standards; Lane 1: Total fraction of non-induced cells; Lane 2: Total fraction of induced cells; Lane 3: The supernatant of total fraction of induced cells; Lane 4: The inclusion body of total fraction of induced cells; Lane 5:Purified protein by using Ni-affinity column.
(TIF)

S3 Fig Comparison of the deduced amino acid sequence of *Ha*VgR with *Sli*VgR, *Ase*VgR, *Ape*VgR and *Bmo*VgR. The cysteine residues are shown with dark-shaded frames. The two clusters of ligand binding repeats (class A repeats) are boxed. The epidermal growth factor (EGF)-like repeats (class B repeats) are underlined. YWXD or potentially related sequences present in class C repeats (YWTD β-propeller domain) are shown with dark-shaded frames. The identical residues are shown with light-shaded frames. The potential transmembrane helix is underlined with ripple. Possible phosphorylation sites are shown with blue-shaded frames. Possible glycosylation sites are shown with gray-shaded frames. The NPXY (with missing tyrosine ‘Y’ residue) internalization signalsare shown with brown-shaded frames.
(TIF)

S1 Table Primers used for identification and analysis of the *HaVgR*. (DOC)

S2 Table Sequence information used for the construction of molecular phylogenetic tree. (DOC)
