# Supplementary figures and images for: Molecular Characterization and Function Analysis of the Vitellogenin Receptor from the Cotton Bollworm, Helicoverpa armigera (Hübner) (Lepidoptera, Noctuidae)
Source: PLoS One. 2016 May 18;11(5):e0155785. doi: 10.1371/journal.pone.0155785 (PMC4871585; doi:10.1371/journal.pone.0155785)

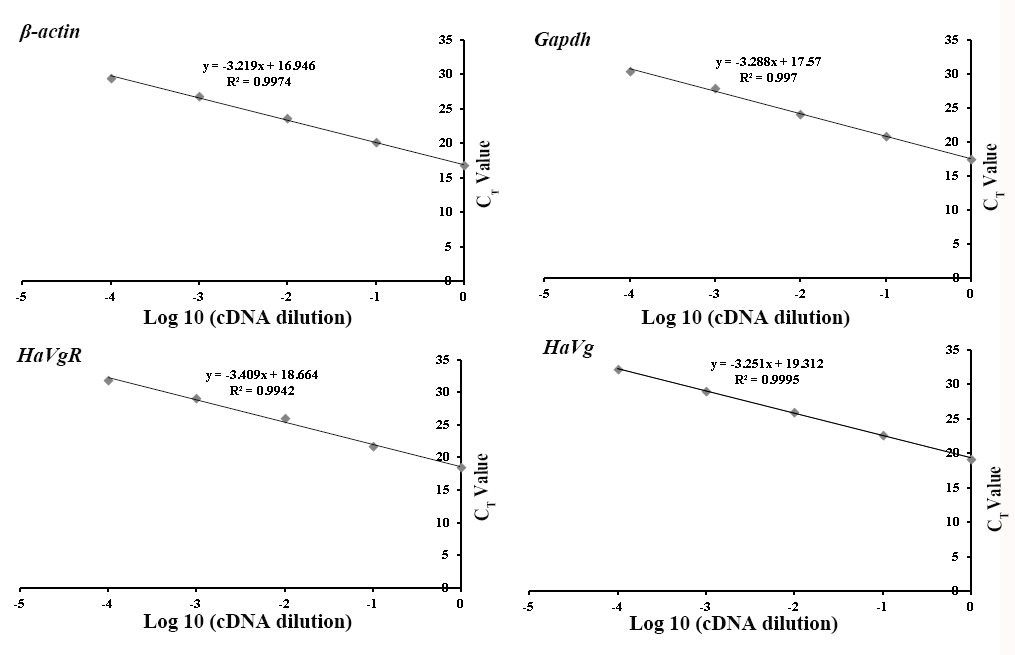

Supplement: S1 Fig — The Ct was calculated for each cDNA dilution. The amplification efficiency of β-actin, Gapdh, HaVgR and HaVg was 107.27%, 101.43%, 96.49% and 103.04%, respectively. (TIF) [file pone.0155785.s001.tif]

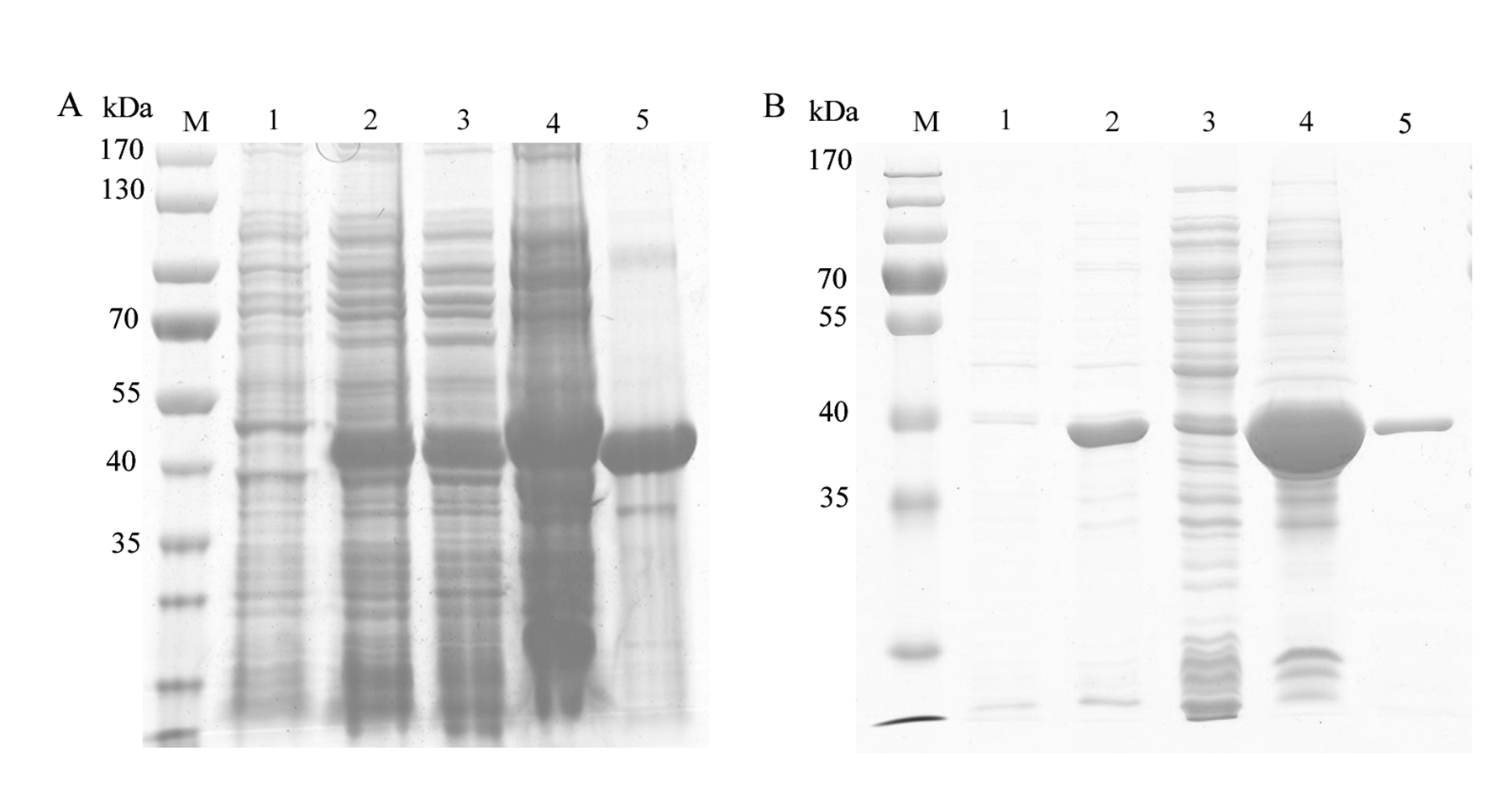

Supplement: S2 Fig — SDS-PAGE analyses showing the expression and purification of recombinant HaVgR (A) and HaVg (B). M: Protein standards; Lane 1: Total fraction of non-induced cells; Lane 2: Total fraction of induced cells; Lane 3: The supernatant of total fraction of induced cells; Lane 4: The inclusion body of total fraction of induced cells; Lane 5:Purified protein by using Ni-affinity column. (TIF) [file pone.0155785.s002.tif]

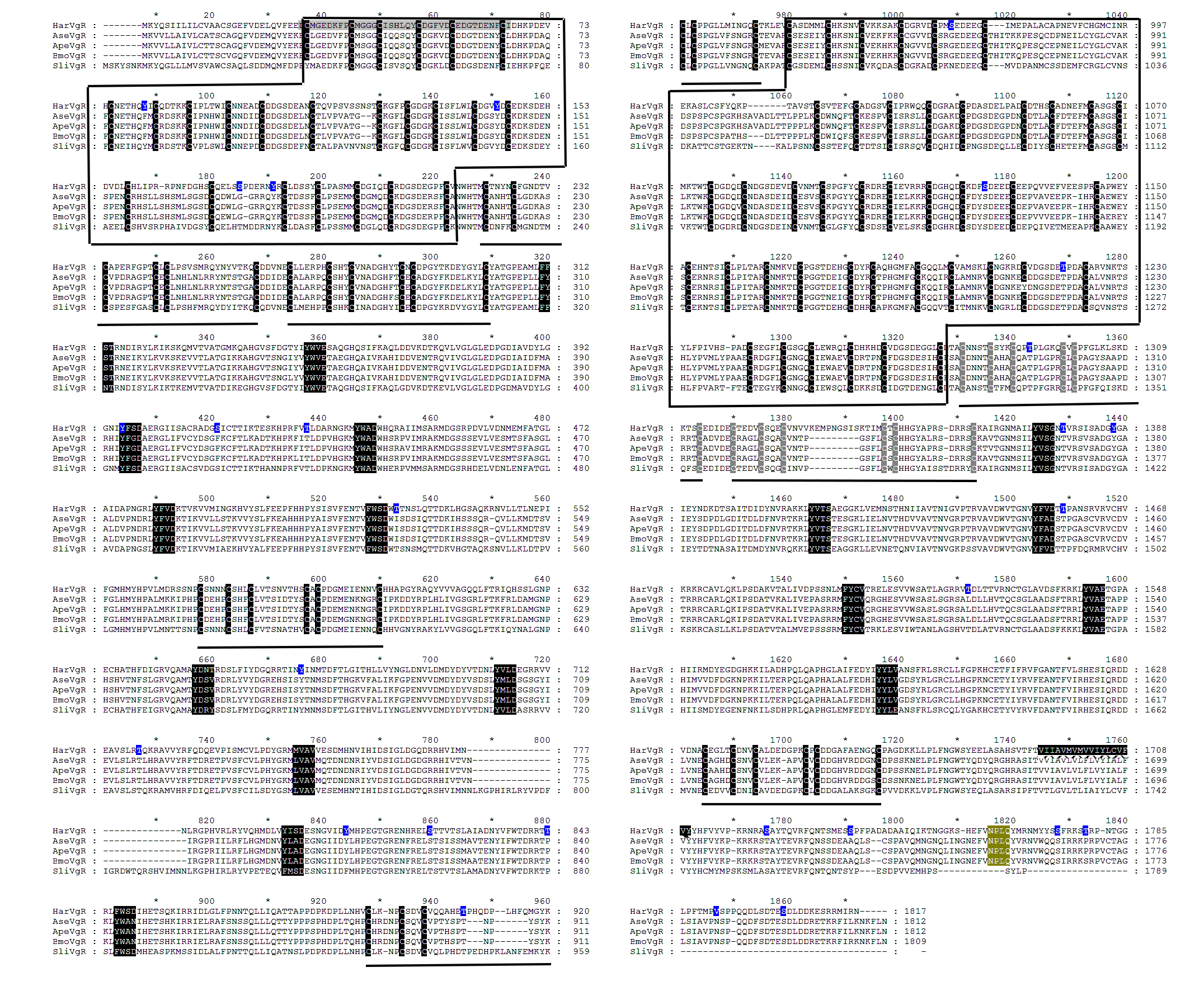

Supplement: S3 Fig — The cysteine residues are shown with dark-shaded frames. The two clusters of ligand binding repeats (class A repeats) are boxed. The epidermal growth factor (EGF)-like repeats (class B repeats) are underlined. YWXD or potentially related sequences present in class C repeats (YWTD β-propeller domain) are shown with dark-shaded frames. The identical residues are shown with light-shaded frames. The potential transmembrane helix is underlined with ripple. Possible phosphorylation sites are shown with blue-shaded frames. Possible glycosylation sites are shown with gray-shaded frames. The NPXY (with missing tyrosine ‘Y’ residue) internalization signalsare shown with brown-shaded frames. (TIF) [file pone.0155785.s003.tif]
