# Supplementary material for: Molecular Characterization and Function Analysis of the Vitellogenin Receptor from the Cotton Bollworm, Helicoverpa armigera (Hübner) (Lepidoptera, Noctuidae)
Source: PLoS One. 2016 May 18;11(5):e0155785. doi: 10.1371/journal.pone.0155785 (PMC4871585; doi:10.1371/journal.pone.0155785)
Supplement: S1 Table — (DOC) [file pone.0155785.s004.doc]

**S1 Table.** **Primers used for identification and analysis of the *HaVgR***

|  |
| --- |

| **Purpose** | **Description** | **Sequence (5'-3')** |
| --- | --- | --- |
| | Splicing  primer | | --- | | HaVgR-F1 | CAAACTACAACTGTTTCGGC |
|  | HaVgR-R1 | GATAAATAGCGAATCTCTAG |
|  | HaVgR-F2 | CATGGACTTGGTGTACATATC |
|  | HaVgR-R2 | AGGGGTCTCATCAGAGCC |
| RACE | HaVgR 5RACE-1 | TCGCACAGCCACAGGAACGAGATG |
|  | HaVgR 5RACE-2 | TGCCGTCGAATGATACGCCGTGAG |
|  | HaVgR 3RACE-1 | CGAGAACTGGAGTCCGTGGTCTGGTC |
|  | HaVgR 3RACE-2 | GACTGCCCCGGCAGTACGGATGAG |
|  | UPM-long | CTAATACGACTCACTATAGGGCAAGCAGTGGTATCAACGCAGAGT |
|  | UPM-short | CTAATACGACTCACTATAGGGC |
| qPCR | HaVgR-F  HaVgR-R  Probe | CCTGCAACAACAGCACATGTT  AAGGGACAGACGCATTGCTT  TACAAGTGCCAGCCGACTCCCCTAGG |
|  | HaVg-F  HaVg-R  Probe | ATGGCGTACTGATGCTCAACAT  GTACCAATCGCTGTCCATCGT  ATGGCCGCCTCCGCGCT |
|  | β-actin-F  β-actin-R  Probe | CTGGGACGATATGGAGAA  CGAACATGATCTGTGTCA  CACCACACCTTCTACAACGAGC |
|  | Gapdh-F  Gapdh-R  Probe | CATTGAAGGTCTGATGACCACTGT  CAGAGGGTCCATCCACTGTCTT  CACGCCACCATTGCCACCCA |
| Protein expression | HaVgR-F  HaVgR-R | CGGGATCCGAAGACGGGACCGATGAAAA  CCCTCGAGTTATGATACGCCGTGAGCCTG |
|  | HaVg-F  HaVg-R | CGGGATCCATTGGTGCTTCATTCTCCGA  CCCTCGAGTTAGTCCTTCCTCTTGTCGGC |
| RNAi | T7 VgR-F  T7 VgR-R | TAATACGACTCACTATAGGGATGAAGACATGGACTTGCGACGG  TAATACGACTCACTATAGGGAGCAGAGGAAGCCCTCGCTGCA |
|  | T7 GFP-F  T7 GFP-R | TAATACGACTCACTATAGGGGCAACATACGGAAAACTTACC  TAATACGACTCACTATAGGGTGTGTGGACAGGTAATGGTTG |
