# Supplementary material for: Molecular Characterization and Function Analysis of the Vitellogenin Receptor from the Cotton Bollworm, Helicoverpa armigera (Hübner) (Lepidoptera, Noctuidae)
Source: PLoS One. 2016 May 18;11(5):e0155785. doi: 10.1371/journal.pone.0155785 (PMC4871585; doi:10.1371/journal.pone.0155785)
Supplement: S2 Table — (DOC) [file pone.0155785.s005.doc]

**S2 Table. Sequence information used for the construction of molecular phylogenetic tree.**

| **Name** | **Species** | **Accession number** |
| --- | --- | --- |
| HaVgR  SliVgR | *Helicoverpa armigera*  *Spodoptera litura* | AGF33811.1  ADK94033.1 |
| AseVgR | *Actias selene* | AFV32171.1 |
| ApeVgR | *Antheraea pernyi* | AEJ88360.1 |
| BmoVgR | *Bombyx mori* | ADK94452.1 |
| DplVgR | Danaus plexippus | EHJ76019.1 |
| NviVgR | *Nasonia vitripennis* | XP_001602954.2 |
| PamVgR | *Periplaneta americana* | BAC02725.2 |
| BgeVgR | *Blattella germanica* | CAJ19121.1 |
| AaeVgR | *Aedes aegypti* | AAK15810.1 |
| MroVgR | *Megachile rotundata* | XP_003704660.1 |
| AgaVgR | *Anopheles gambiae* | EAA06264.6 |
| RmaVgR | *Rhyparobia maderae* | BAE93218.1 |
| BdoVgR | *Bactrocera dorsalis* | AGE83235.1 |
| AflVgR | *Apis florea* | XP_003690500.1 |
| HsaVgR | *Harpegnathos saltator* | EFN84770.1 |
| BteVgR | *Bombus terrestris* | XP_003402703.1 |
| DgrVgR | *Drosophila grimshawi* | GH17685 |
| CcaVgR | *Ceratitis capitata* | XP_004529643.1 |
| DmoVgR | *Drosophila mojavensis* | GI21507 |
| DerVgR | *Drosophila erecta* | GG17831 |
| NluVgR | Nilaparvata lugens | ADE34166 |
| ApisLDLR | *Acyrthosiphon pisum* | XP_001944152.2 |
| DmeVgR | *Drosophila melanogaster* | AAB60217.1 |
| AmeVgR | *Apis mellifera* | XP_001121707.2 |
| PhuVgR | *Pediculus humanus corporis* | XP_002423121.1 |
| BimVgR | *Bombus impatiens* | XP_003489577.1 |
| SinVgR | *Solenopsis invicta* | AAP92450.1 |
| MdoVgR | *Musca domestica* | XP_005190432.1 |
| AheVgR | *Amblyomma hebraeum* | AGQ57038.1 |
| CflVgR | *Camponotus floridanus* | EFN61730.1 |
| DvaVgR | *Dermacentor variabilis* | AAZ31260.3 |
| HloVgR | *Haemaphysalis longicornis* | BAG14342.1 |
| TcaVgR | *Tribolium castaneum* | XP_968903.2 |
| PmoVgR | *Penaeus monodon* | ABW79798.1 |

S2 Table (Continue)

| **Name** | **Species** | **Accession number** |
| --- | --- | --- |
| MroVgR, | *Macrobrachium rosenbergii* | ADK55596.1 |
| AcaLDLR | *Aplysia californica* | XP_005090980.1 |
| XmaLDLR | *Xiphophorus maculatus* | XP_005799449.1 |
| AmeLDLR | *Ailuropoda melanoleuca* | XP_002916562.1 |
| PhoLDLR | *Pantholops hodgsonii* | XP_005977011.1 |
| BtaLDLR | *Bos taurus* | DAA32788.1 |
| OniLDLR | *Oreochromis niloticus* | XP_005453016.1 |
| PabLDLR | *Pongo abelii* | XP_002812613.1 |
| HbuLDLR | *Haplochromis burtoni* | XP_005918229.1 |
| Hsa LDLR | *Homo sapiens* | EAX11280.1 |
